# Electrosensory Midbrain Neurons Display Feature Invariant Responses to Natural Communication Stimuli

**DOI:** 10.1371/journal.pcbi.1004430

**Published:** 2015-10-16

**Authors:** Tristan Aumentado-Armstrong, Michael G. Metzen, Michael K. J. Sproule, Maurice J. Chacron

**Affiliations:** 1 School of Computer Science, McGill University, Montreal, Quebec, Canada; 2 Department of Physiology, McGill University, Montreal, Quebec, Canada; The Krasnow Institute for Advanced Studies, UNITED STATES

## Abstract

Neurons that respond selectively but in an invariant manner to a given feature of natural stimuli have been observed across species and systems. Such responses emerge in higher brain areas, thereby suggesting that they occur by integrating afferent input. However, the mechanisms by which such integration occurs are poorly understood. Here we show that midbrain electrosensory neurons can respond selectively and in an invariant manner to heterogeneity in behaviorally relevant stimulus waveforms. Such invariant responses were not seen in hindbrain electrosensory neurons providing afferent input to these midbrain neurons, suggesting that response invariance results from nonlinear integration of such input. To test this hypothesis, we built a model based on the Hodgkin-Huxley formalism that received realistic afferent input. We found that multiple combinations of parameter values could give rise to invariant responses matching those seen experimentally. Our model thus shows that there are multiple solutions towards achieving invariant responses and reveals how subthreshold membrane conductances help promote robust and invariant firing in response to heterogeneous stimulus waveforms associated with behaviorally relevant stimuli. We discuss the implications of our findings for the electrosensory and other systems.

## Author Summary

We provide the first experimental evidence showing that midbrain electrosensory neurons in the weakly electric fish species *Apteronotus leptorhynchus* can respond in an invariant manner to the heterogeneous stimulus waveforms associated with natural electrocommunication stimuli. Interestingly, hindbrain neuron populations providing afferent input did not display feature invariant responses. In order to understand the mechanisms that mediate the emergence of feature invariance in midbrain neurons, we built a model based on the Hodgkin-Huxley formalism. An evolutionary algorithm identified multiple combinations of parameter values that all gave rise to responses that matched those seen experimentally. In particular, balanced input from ON and OFF-type cells was necessary to observe feature invariance. Moreover, while a spiking nonlinearity was sufficient to observe invariant responses, the addition of subthreshold membrane conductances to the model enhanced the regions in parameter space for which feature invariant responses were observed. Further analysis of membrane potential responses confirmed our modeling predictions. Our model thus makes the important prediction that multiple mechanisms will lead to feature invariance and we discuss the implications for sensory processing in the electrosensory and other systems.

## Introduction

Efficient processing of incoming sensory information is essential to an organism’s survival. Thus, understanding the strategies used by the brain to process such information (the neural code) remains an important problem in neuroscience. There is growing experimental evidence that the representation of sensory information changes from a dense code, in which neurons respond differentially to most if not all behaviorally relevant stimuli, to a sparse code, in which neurons instead respond selectively but in an invariant manner to a given stimulus (e.g. a human face) [1–12]. This is thought to reduce energy consumption, enhance the ability to recognize a particular feature, and facilitate readout by higher brain structures [13].

Despite their seemingly contrary nature, both response selectivity and invariance to identity preserving transforms of the target stimuli (e.g. the same object seen from different viewpoints or the same sound heard at different intensities) have been shown to progressively increase as information propagates to higher brain regions [8, 10–12, 14]. Most strikingly, neurons in the medio-temporal cortex can respond similarly to stimulus patterns that are only abstractly related (a picture of a given person vs. the name of this person written on a piece of paper) [6]. The mechanisms by which sparse coding and feature invariance emerge in the vertebrate brain remain largely unknown but are critical for understanding brain disorders as well as improving artificial intelligence [15].

Here we studied the emergence of feature invariant representations of natural stimuli in the electrosensory system of the gymnotiform weakly electric fish *Apteronotus leptorhynchus*. These animals continuously emit a quasi-sinusoidal electric field through an electric organ discharge (EOD) and rely on perturbations of this field caused by objects whose conductivity is different than that of the surrounding water (e.g. prey, conspecifics) to obtain information about their surroundings (see [16–21] for review). Peripheral electroreceptor neurons respond to increases in EOD amplitude through increases in firing rate and relay this information to pyramidal neurons within the hindbrain electrosensory lateral line lobe (ELL) that in turn project to the midbrain torus semicircularis (TS), which is at a similar processing stage as the inferior colliculus in mammals [17]. Natural stimuli are comprised in part by those caused by conspecifics. In particular, the interference between the EODs of two fish that come into contact (<1 m distance) will give rise to a sinusoidal amplitude modulation stimulus, a beat, whose frequency is equal to the difference between the two EOD frequencies. Due to a sexual dimorphism in EOD frequency, interactions between same-sex and opposite-sex conspecifics give rise to low (<30 Hz) and high (>30 Hz) frequency beats, respectively [22]. These fish moreover will display electrocommunication stimuli called “chirps” that consist of brief (<40 ms) increases in EOD frequency, which occur on top of the beat and can give rise to very different waveforms [22–26].

Previous electrophysiological studies have found that both peripheral electroreceptors [27–29] as well as hindbrain pyramidal neurons [30–32] tend to use dense neural codes as they give strong responses to both beat and chirp stimuli that vary based on the waveform used. In contrast, TS neurons tend to use a sparse neural code as they display much more selectivity. In particular, some TS neurons can respond selectively but differentially to chirps [32]. Here we report that some TS neurons can instead respond selectively but in an invariant fashion to different chirp waveforms, thereby providing a potential neural correlate of robust perception of natural electrocommunication signals by permitting reliable signal detection and information extraction in higher brain areas. Since hindbrain ELL neurons providing afferent input to TS neurons did not display such invariant responses, we concluded that feature invariance emerges at the level of the TS presumably by nonlinear integration of ELL afferent input. In order to investigate the underlying mechanisms, we built a model of a TS neuron receiving realistic input based on the Hodgkin-Huxley formalism. Our results show that multiple combinations of parameter values could give rise to feature invariant responses to different chirp waveforms mimicking those seen experimentally. Importantly, while a spiking nonlinearity was sufficient to observe such responses, the addition of subthreshold h- and T-type currents increased the regions in parameter space that gave rise to feature invariance. Our results have important implications for understanding the emergence of feature invariant responses in sensory systems.

## Results

**Fig 1:**
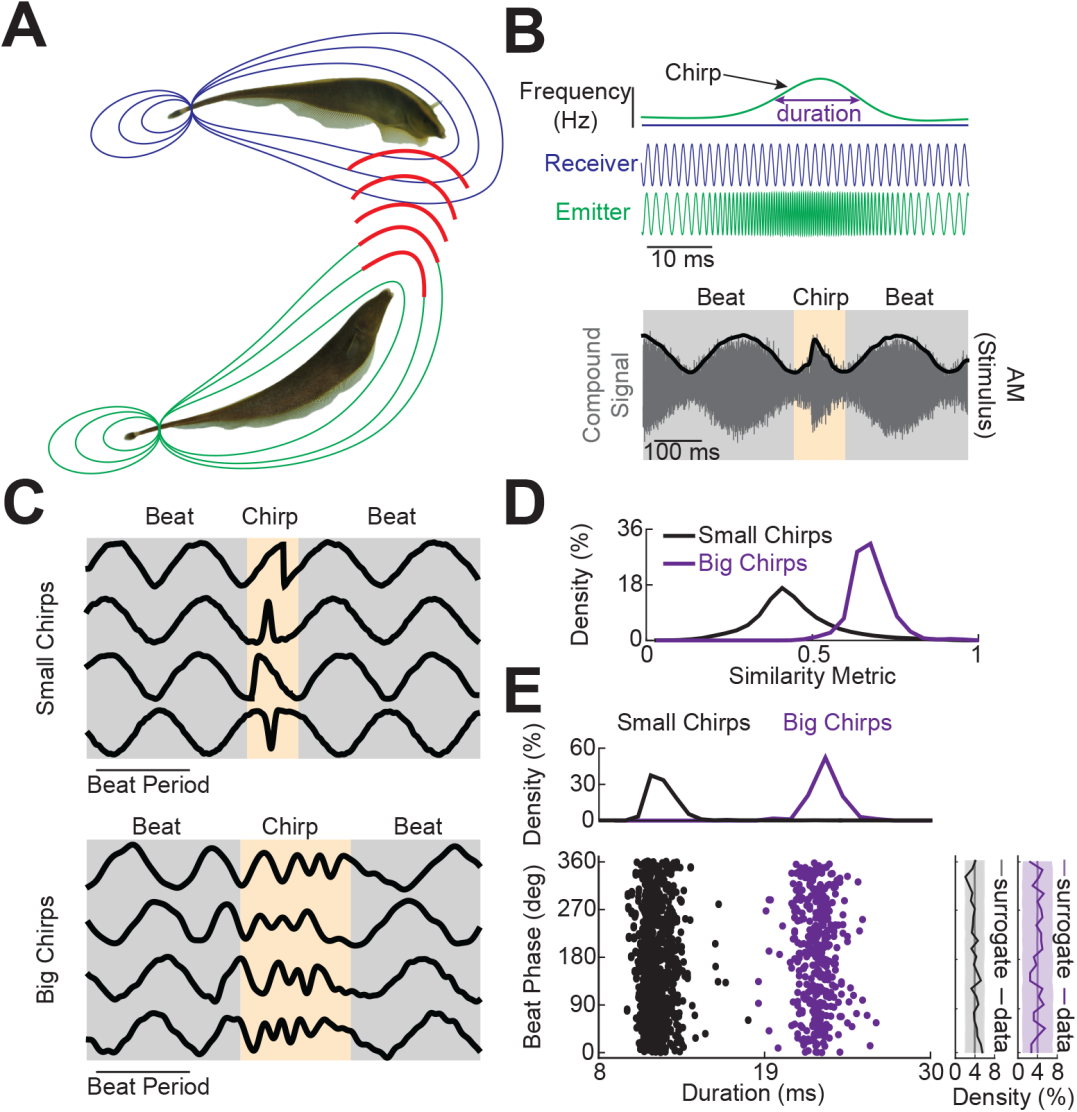
Natural electrocommunication stimuli display heterogeneous waveforms. **A)** Two weakly electric fish with their electric organ discharges (EODs) and a communication signal (“chirp”) from the emitter fish (green) to the receiver fish (blue). **B)** Schematic showing the EODs of the emitter and receiver (middle) fish as a function of time as well as the instantaneous EOD frequencies (top). The chirp consists of a transient increase in the emitter fish’s EOD frequency while the receiver fish’s frequency remains constant. The compound signal (bottom, gray) then consists of a beat resulting from the interference between the two quasi-sinusoidal EOD waveforms and the chirp then leads to a brief interruption in the beat. Note that the actual stimulus is the AM of the compound signal (bottom, black). **C)** Example waveforms resulting from natural chirping behavior. Note the large heterogeneities in the waveforms associated with the small (top) but not big chirps (bottom). **D)** Probability distribution of the similarity measure for big and small chirps. **E)** Chirp stimuli occur on all phases of the beat with uniform probability density but have fixed durations.

Our study focuses on how the electrosensory system of weakly electric fish can give rise to invariant neural responses to heterogeneous waveforms associated with natural electrocommunication stimuli. Such stimuli occur during interactions between two individual fish (Fig 1A). Because each individual fish has a different EOD frequency, interaction between the quasi-sinusoidal waveforms gives rise to a beat (Fig 1B, black trace in bottom panel) [22, 25]. Electrocommunication stimuli (i.e. “chirps”) consist of a brief (<40 ms) increase in the emitter fish’s EOD frequency (Fig 1B, green trace in top panel) while the EOD frequency of the receiver fish remains constant (Fig 1B, blue trace in top panel). Chirps have been traditionally segregated into type I (“big”) and type II (“small”): big chirps consist of a large increase in frequency (>150 Hz) accompanied by a decrease in the emitter fish’s EOD amplitude and tend to occur for large (>30 Hz) beat frequencies while small chirps instead consist mostly of a smaller (>30 Hz and <150 Hz) increase in the EOD frequency of the emitter fish that tend to occur for small (<30 Hz) beat frequencies [22, 23, 33].

### Natural electrocommunication stimuli display heterogeneities

While it is well known that natural electrocommunication stimuli display heterogeneous waveforms [25, 27], a systematic investigation and quantification of these heterogeneities has not been done to date. Thus, we first investigated and quantified heterogeneities in naturally occurring electrocommunication stimuli. We used a well-established behavioral assay [34] in which the restrained animal receives a stimulus mimicking another fish’s EOD and recorded the animal’s behavioral responses (see Methods). On average the EOD frequency increases associated with small and big chirps were 50.1±0.5 Hz (min: 30.1 Hz; max: 76.5 Hz; n = 486) and 261.2±2.2 Hz (min: 155.4 Hz; max: 459.3 Hz; n = 395), respectively. Overall, we found large differences between the waveforms associated with small chirps as evidenced from the four example traces shown in the top panel of Fig 1C. In contrast, we found smaller differences amongst the waveforms associated with big chirps that were all relatively similar to each other as they were mainly characterized by a pronounced decrease in the beat amplitude (Fig 1C, bottom panel). We quantified the difference between the observed small chirp and big chirp waveforms using a similarity index (see Methods) and found overall larger values for big chirps than for small chirps (two-sample K-S test, p = 0.0013) (Fig 1D).

In order to investigate the source of the observed heterogeneities in the recorded small chirp but not big chirp waveforms, we quantified each by two attributes: namely duration and beat phase (see Methods). Our results show that both small and big chirp duration were distributed over different but relatively narrow ranges of values (Fig 1E). In contrast, both small and big chirps occurred at all phases of the beat with uniform probability (Fig 1E). Thus, our results suggest that the phase of the beat at which the chirp occurs but not its duration is an important source of heterogeneity in the resulting stimulus waveform for small but not big chirps. This result can be understood intuitively as follows. First, we note that small and big chirps tend to occur on top of lower and higher frequency beats, respectively. Thus, the beat period is then longer relative to the chirp duration for the former and is thus expected to have more of an effect in creating a heterogeneous set of waveforms. We nevertheless note that the fact that there is a concomitant decrease in EOD amplitude for big but not small chirps also likely contributes to the fact that there are less heterogeneities in the waveforms resulting from the former.

### TS neurons display invariant responses to electrocommunication stimuli

**Fig 2:**
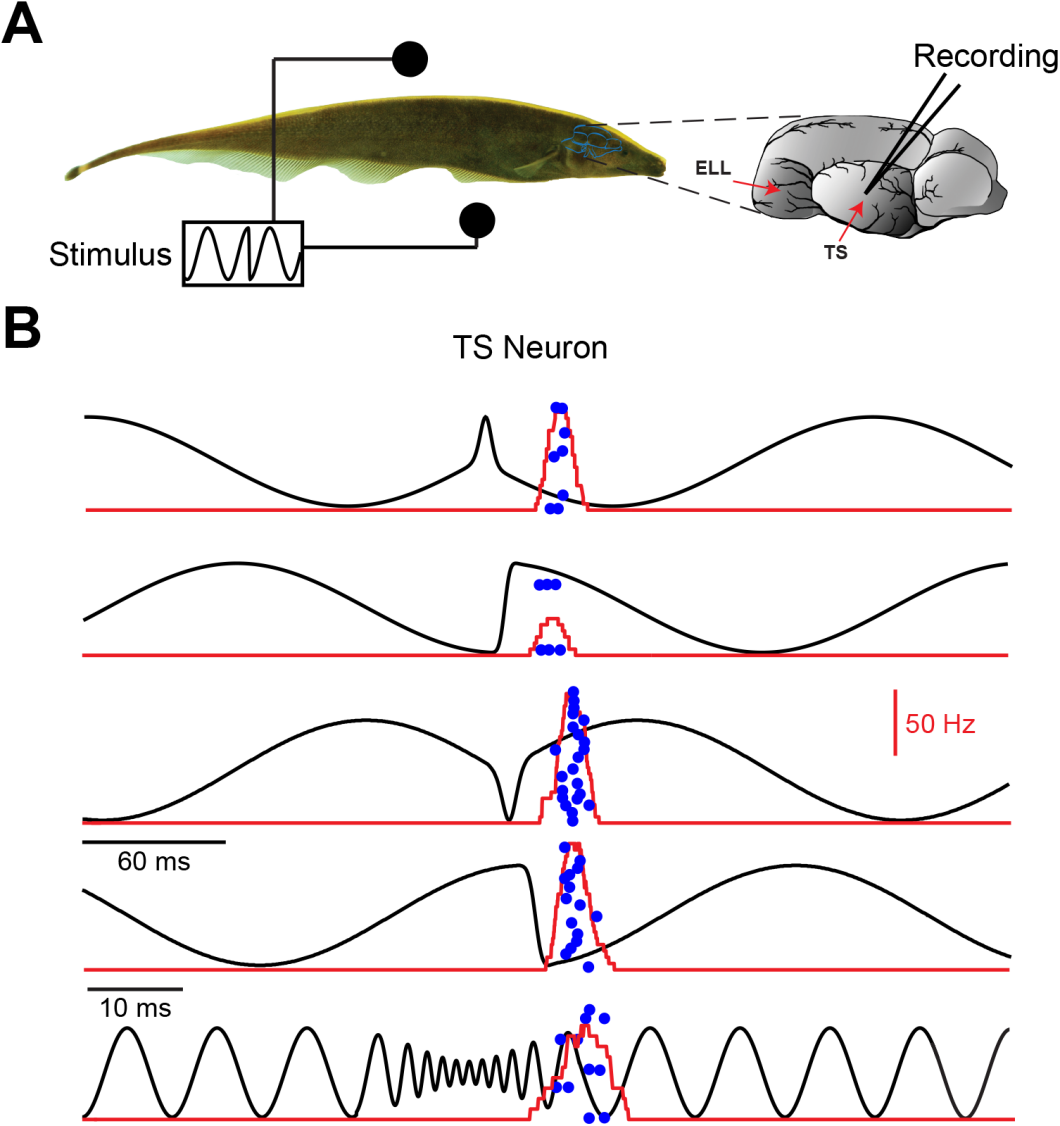
Midbrain electrosensory neurons display feature invariant responses to natural electrocommunication stimuli. **A)** Schematic showing a fish with stimulation electrodes on each side as well as a recording electrode from the midbrain region Torus semicircularis (TS). **B)** Responses of an example TS neuron to different chirp stimuli (black). The blue dots mark the occurrence of action potentials to 20 repeated presentations of the stimulus waveform (raster plot) and the red curve shows the firing rate response averaged over trials (PSTH). Note the similarity in the response to each waveform as the neuron fires at the chirp onset with one-two spikes in each case.

We next investigated whether electrosensory midbrain neurons responded to big and small chirps in a feature invariant manner. To do so, we recorded the responses of TS neurons (N = 137) (Fig 2A) to both small and big chirp stimuli that captured the relatively heterogeneous waveforms seen for the former and the relatively homogeneous waveforms seen for the latter (Fig 2B). Previous studies have found that TS neurons either do not respond selectively or respond selectively but differentially to different chirp stimulus waveforms [32]. Here we focused on neurons that responded selectively to the chirp stimulus and not the beat and whose responses to different chirp waveforms were similar (see Methods). We found that some TS neurons in our dataset (N = 9) responded selectively to both small and big chirp stimuli but in a similar manner through silence during the beat and by the firing of 1–2 action potentials at a short latency (~15 ms) after the chirp onset (Fig 2B). We quantified whether the response was selective to the chirp waveform using the chirp selectivity index (CSI) as done previously [32] (see Methods) and we quantified differences between spiking responses to different chirp waveforms using the Victor-Purpura distance metric (VPD) [35] (see Methods). We obtained CSI = 1 and VPD = 1.19 for the example neuron shown in Fig 2. We also computed a feature invariance index (FI) score that captured a neuron’s ability to respond selectively but invariantly to chirps. We obtained FI = 0.99 for the example neuron shown in Fig 2.

### ELL pyramidal neurons do not display invariant responses to electrocommunication stimuli

**Fig 3:**
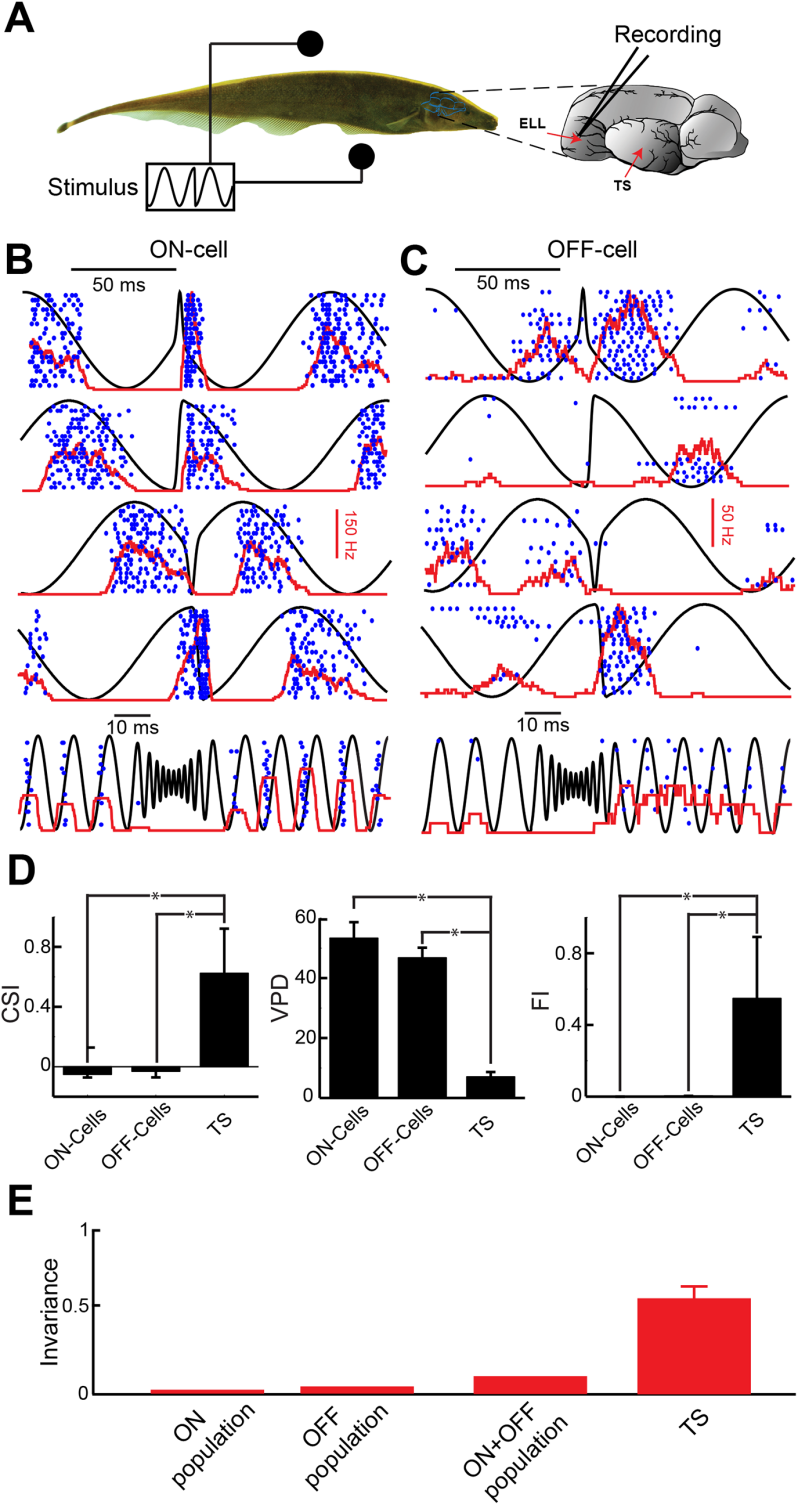
Hindbrain electrosensory neurons projecting to midbrain do not display feature invariant responses to natural electrocommunication stimuli. **A)** Schematic showing a fish with stimulation electrodes on each side as well as a recording electrode from the hindbrain region Electrosensory lateral line lobe (ELL). **B,C)** Responses of example ON and OFF-type ELL pyramidal neurons to different chirp stimuli (black), respectively. The blue dots mark the occurrence of action potentials to 20 repeated presentations of the stimulus waveform (raster plot) and the red curve shows the firing rate response averaged over trials (PSTH). Note the different responses to chirps as a given cell responds sometimes with excitation and sometimes with inhibition. **D)** Population-averaged VPD (left), CSI (middle), and FI (right) values for ELL ON, OFF-cells, and TS neurons. “*” indicates statistical significance at the p = 0.05 level using a one-way ANOVA. Values for ON, OFF, and TS cells, respectively, are VPD = 53.3±5.3, 46.8±3.4, 7.4±1.9; CSI = -0.05±0.02, -0.03±0.04, 0.61±0.08; FI = 0.00±0.00, 0.004±0.004, 0.536±0.093. **E)** FI values obtained from the population-averaged responses for ON-cells, OFF-cells, and ON+OFF-cells are also shown and are all much lower than FI values obtained for TS neurons.

Perhaps the simplest potential explanation for the experimentally observed invariant responses of TS neurons described above is that they are simply inherited from their afferent ELL pyramidal neurons. Previous studies have found two types of ELL pyramidal neurons [36]: ON-type neurons respond with excitation while OFF-type neurons instead respond with inhibition to increases in EOD amplitude, respectively. We thus recorded ELL pyramidal neuron responses to the same stimuli presented to TS neurons (Fig 3A). In contrast to TS neurons and consistent with previous results [37], ON (n = 25) and OFF-type (n = 20) ELL pyramidal cells displayed pronounced responses to the beat in the form of phase locking (Fig 3B and 3C). Although ON and OFF-type ELL pyramidal cells also responded to all chirp waveforms, they did not do so in an invariant manner as they were excited by some chirp waveforms but inhibited by others (Fig 3B and 3C). We quantified the responses of ON and OFF-type ELL pyramidal cells to chirps using CSI, VPD, and FI. Overall, both ON and OFF-type ELL neurons displayed significantly smaller CSI values than TS neurons (Fig 3D, left) indicating that they tended to respond to both the beat and the chirp, consistent with previous findings [30–32]. Moreover, there were significantly greater differences between the responses of ON and OFF-type ELL neurons to different chirp waveforms as compared to that of TS neurons (Fig 3D, middle). Therefore, the responses of TS neurons to chirps were significantly more invariant than those of ELL neurons (ON: 0.000±0.000, OFF: 0.004±0.004, TS: 0.54±0.09, one-way ANOVA with Tukey-Kramer correction, p<0.05) (Fig 3D, right). We conclude that the feature invariant responses observed in TS are not simply inherited from ELL neurons and must rather result from TS neurons integrating such input.

### Feature invariant responses in TS neurons require nonlinear integration of ELL afferent input

We next tested whether pooling the activities of ON and OFF-type pyramidal cells might give rise to feature invariant responses. This is important because previous results have found strong heterogeneities in the responses of ON and OFF-type pyramidal cells to chirp stimuli [30, 31]. Overall, we found that pooling the responses across either ON-type, OFF-type, or both types did not give rise to feature invariant responses to natural electrocommunication stimuli (Fig 3E). While such pooling gave rise to slightly higher FI values than seen for single neurons, the FI values were still much lower than those observed in TS neurons (Fig 3E). We thus conclude that nonlinear integration of ELL input by TS neurons is necessary to give rise to the experimentally observed feature invariant responses in the midbrain.

### A Hodgkin-Huxley model displays feature invariant responses to natural electrocommunication stimuli

**Fig 4:**
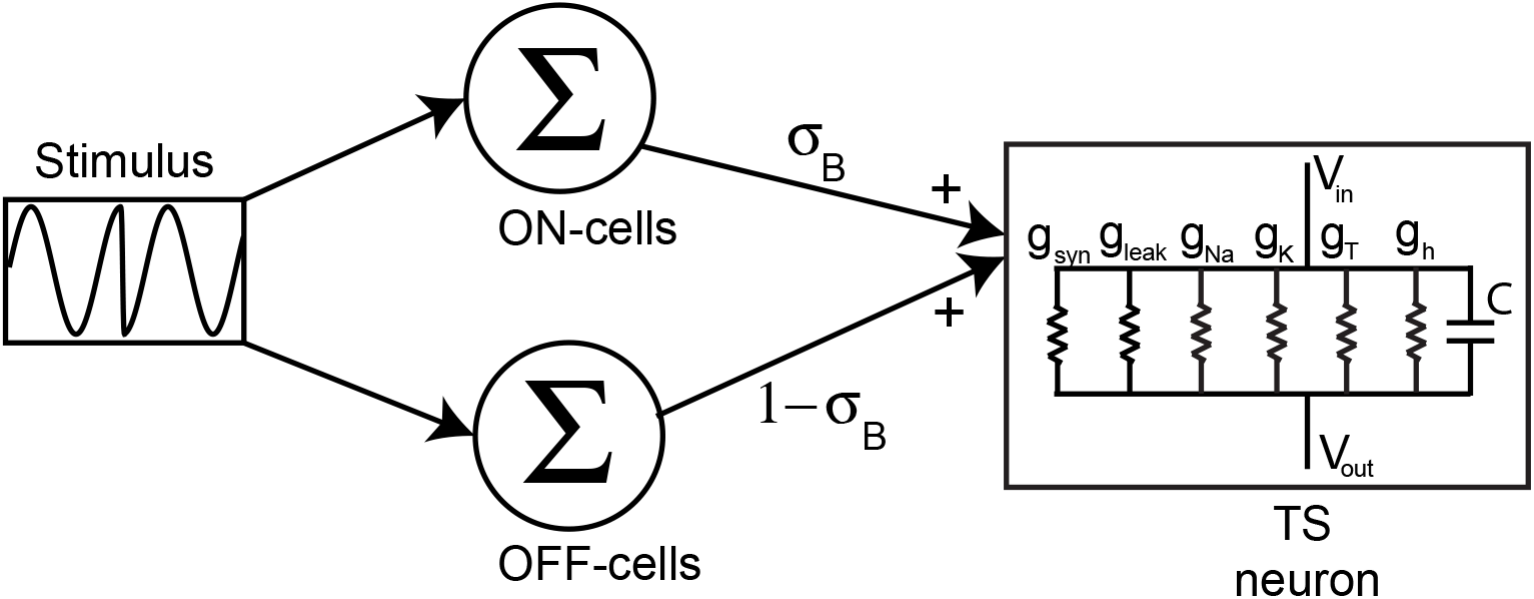
Modeling TS neuron responses to natural electrocommunication stimuli. Our model consists of summing the population-averaged responses of ON and OFF-type ELL pyramidal neurons with weights σ_B_ and 1-σ_B_, respectively, and giving the resulting signal as synaptic input to a model TS neuron that includes various membrane conductances that were modeled using the Hodgkin-Huxley formalism: leak *g*_*leak*_, spiking sodium *g*_*Na*_, delayed rectifier potassium *g*_*K*_, T-type calcium *g*_*T*_, hyperpolarization activated inward rectifier *g*_*h*_.

To investigate whether, and if so how, nonlinear integration of ELL input by TS neurons is sufficient to observe feature invariant responses to chirp stimuli, we built a model TS neuron based on the Hodgkin-Huxley formalism that included different membrane conductances seen experimentally in TS neurons (Fig 4). Importantly, the afferent ELL input to the model was the weighted sum of the population-averaged experimentally observed responses of ON and OFF-type pyramidal neurons to chirp stimuli convolved with an alpha function to mimic synaptic input (see Methods). Model parameters were similar to those used in previous modeling studies of TS neurons [38–40] or varied systematically. We then used a constrained differential evolution algorithm to identify combinations of parameter values that gave rise to the highest feature invariant responses as quantified by FI (see Methods).

**Fig 5:**
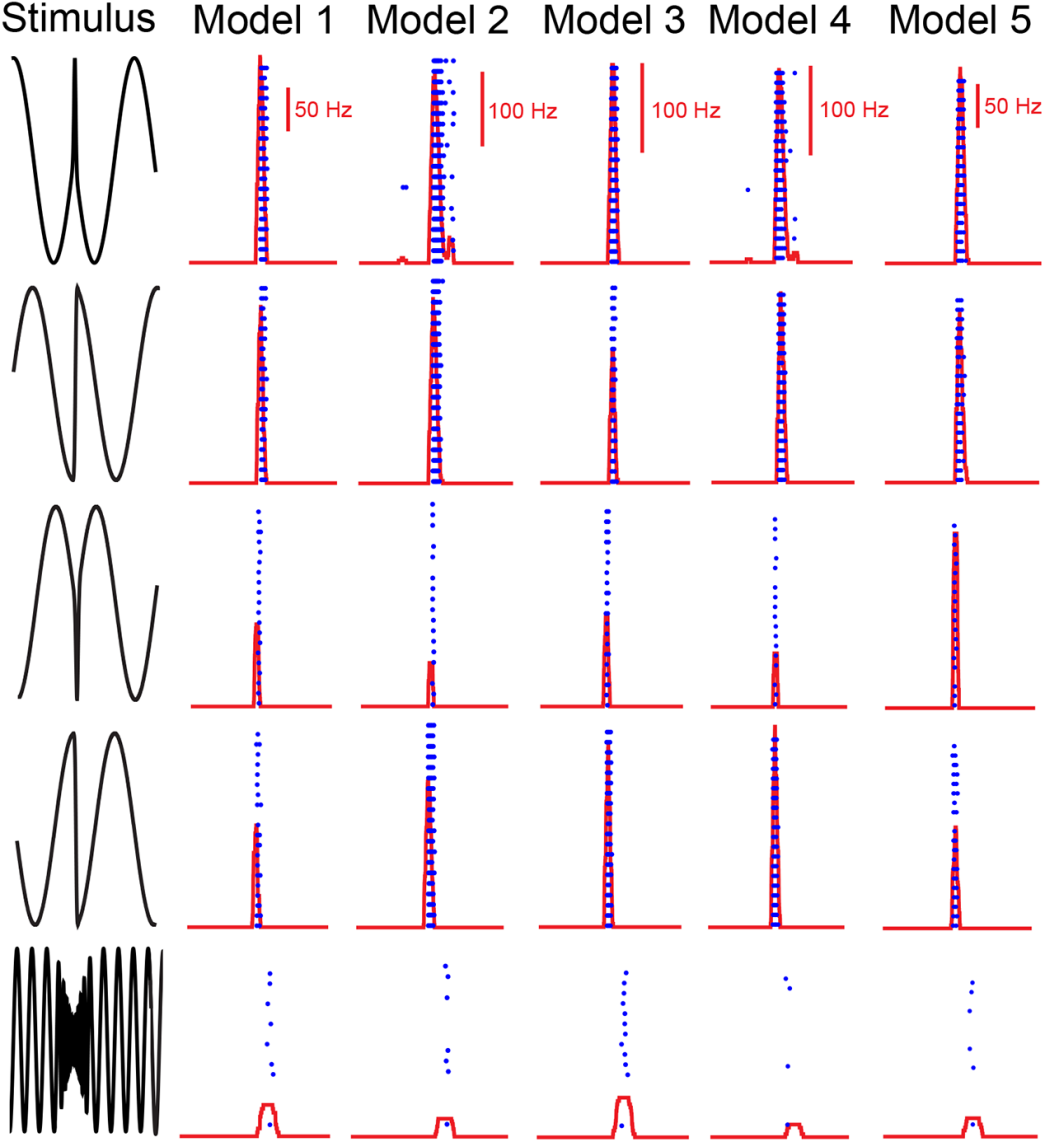
Widely different combinations of parameter values give rise to feature invariant responses in our model. Five example model neurons and their responses to the different chirp stimuli. The FI values for models 1 to 5 were 0.90, 0.79, 0.91, 0.77, and 0.89, respectively. Parameter values for models 1 to 5 where *σ*_*B*_ = 0.42, 0.41, 0.35, 0.40, 0.42; *I*_*bias*_ = -9.4, -6.6, -18.1, -5.2, -6.5 nA; *g*_*syn*_ = 0.10, 0.13, 0.16, 0.10, 0.09 μS; *g*_*h*_ = 0.24, 0, 0.48, 0.02, 0 μS; *g*_*T*_ = 2.10, 0, 3.99, 0, 5.6 μS. Other parameter values were the same as that indicated in the methods.

This algorithm identified multiple sets of physiologically realistic parameter values that all gave rise to feature invariant responses that matched those seen experimentally. Fig 5 shows five such examples. For each set of parameter values, the model neuron responded to each chirp with 1–4 action potentials, as seen experimentally, in a similar fashion as quantified by similar FI scores despite having different parameter values. Importantly, we had both T- and h-type conductances set to zero (i.e. *g*_*T*_ = *g*_*h*_ = 0 μS) for model 2 indicating that these subthreshold membrane conductances are not necessary to observe feature invariance. Thus, our model predicts that the spiking nonlinearity is sufficient to produce feature invariant responses to natural electrocommunication stimuli.

### Effect of varying model parameters on feature invariance

**Fig 6:**
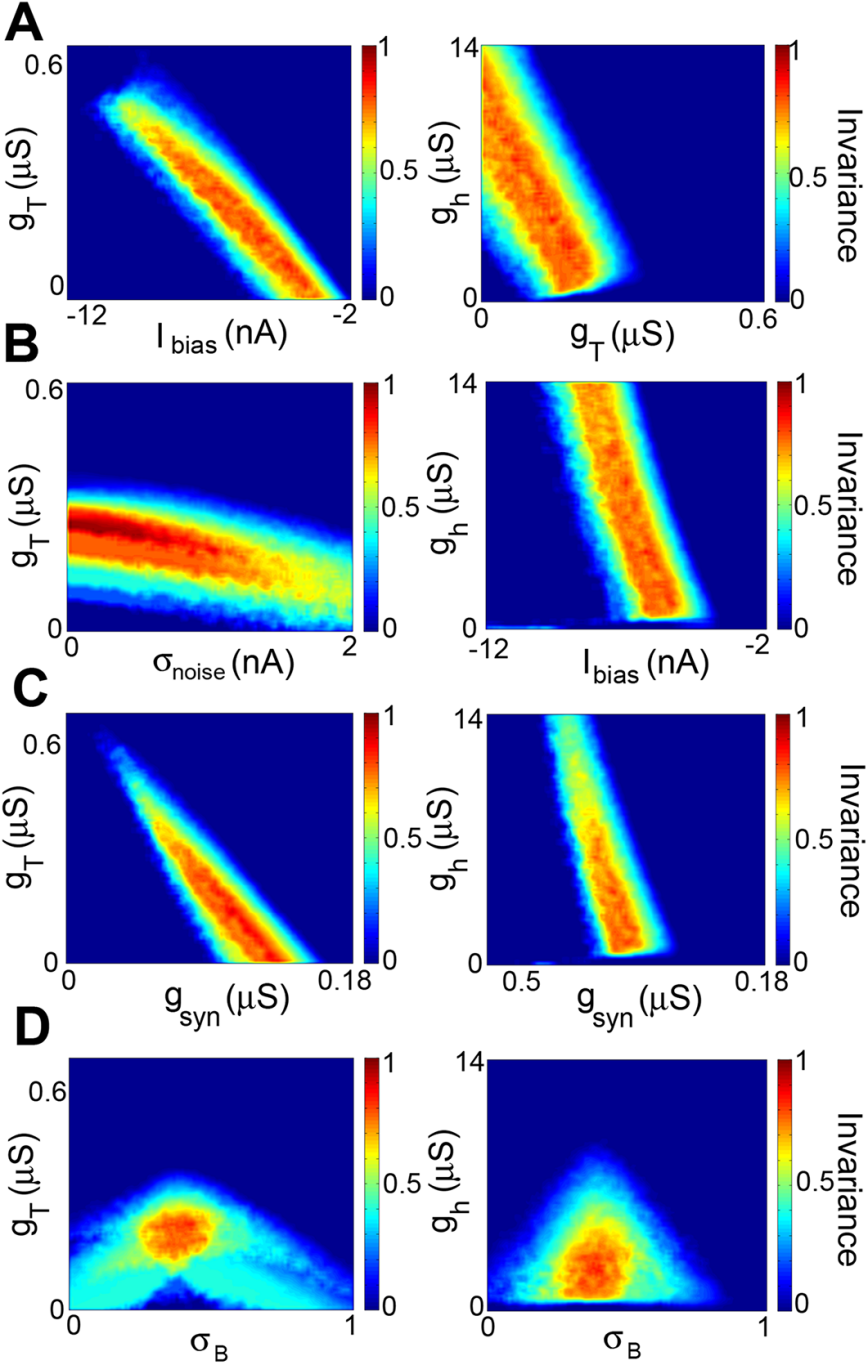
The balance of ON vs. OFF input is critical to observe feature invariance whereas compensation was observed for other parameters. **A)** Invariance as a function of *I*_*bias*_ and *g*_*T*_ (left) and of *I*_*bias*_ and *g*_*h*_ (right). **B)** Invariance as a function of *σ*_*noise*_ and *g*_*T*_ (left) and *σ*_*noise*_ and *g*_*h*_ (right). **C)** Invariance as a function of *σ*_*noise*_ and *g*_*T*_ (left) and *σ*_*noise*_ and *g*_*h*_ (right). **C)** Invariance as a function of *g*_*syn*_ and *g*_*T*_ (left) and *g*_*syn*_ and *g*_*h*_ (right). For A,B,C, note the negatively sloped red band indicating that a decrease/increase in the former can be compensated by an increase/decrease in the latter. **D)** Invariance as a function of *σ*_*B*_ and *g*_*T*_ (left) and *σ*_*B*_ and *g*_*h*_ (right). Note that the highest feature invariance was observed when *σ*_*B*_ ∼ 0.4 in both cases. Other parameter values were *g*_*syn*_ = 0.063 μS, *g*_*h*_ = 2.34 μS, *g*_*T*_ = 0.21 μS, *I*_*bias*_ = –5.72 nA, and *σ*_*B*_ = 0.41.

**Fig 7:**
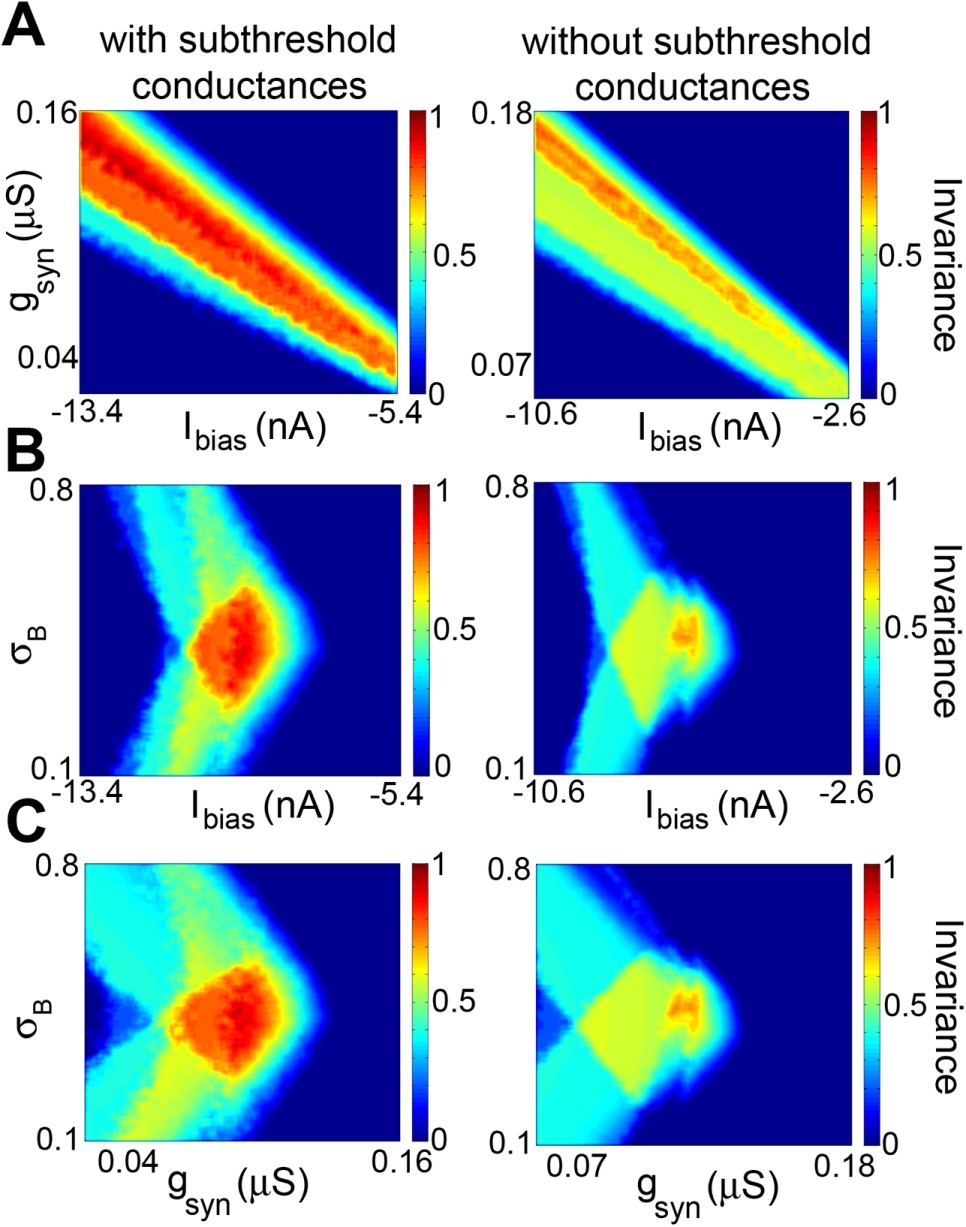
Subthreshold membrane conductances increase the set of parameter values for which feature invariance is observed. **A)** Invariance as a function of *I*_*bias*_ and *g*_*syn*_ with (left) and without (right) the subthreshold membrane conductances *g*_*T*_ and *g*_*h*_. Robustness (i.e. the % of pixels for which the feature invariance score FI was greater of equal to 0.7, see Methods) was 20% (left) vs. 4% (right). **B)** Invariance as a function of *I*_*bias*_ and *σ*_*B*_ with (left) and without (right) the subthreshold membrane conductances *g*_*T*_ and *g*_*h*_. Robustness was 7.8% (left) vs. 0.5% (right). **C)** Invariance as a function of *g*_*syn*_ and *σ*_*B*_ with (left) and without (right) the subthreshold membrane conductances *g*_*T*_ and *g*_*h*_. Robustness was 5.9% (left) vs. 0.3% (right). For the panels on the left, we had *g*_*T*_ = 0.236 μS and *g*_*h*_ = 2.1 μS whereas for those on the right, we had *g*_*T*_ = *g*_*h*_ = 0 μS. Other parameter values were *g*_*syn*_ = 0.063, 0.128 μS; *I*_*bias*_ = –9.39, –6.57 nA; and *σ*_*B*_ = 0.42, 0.41 for the panels on the left and right columns, respectively.

**Fig 8:**
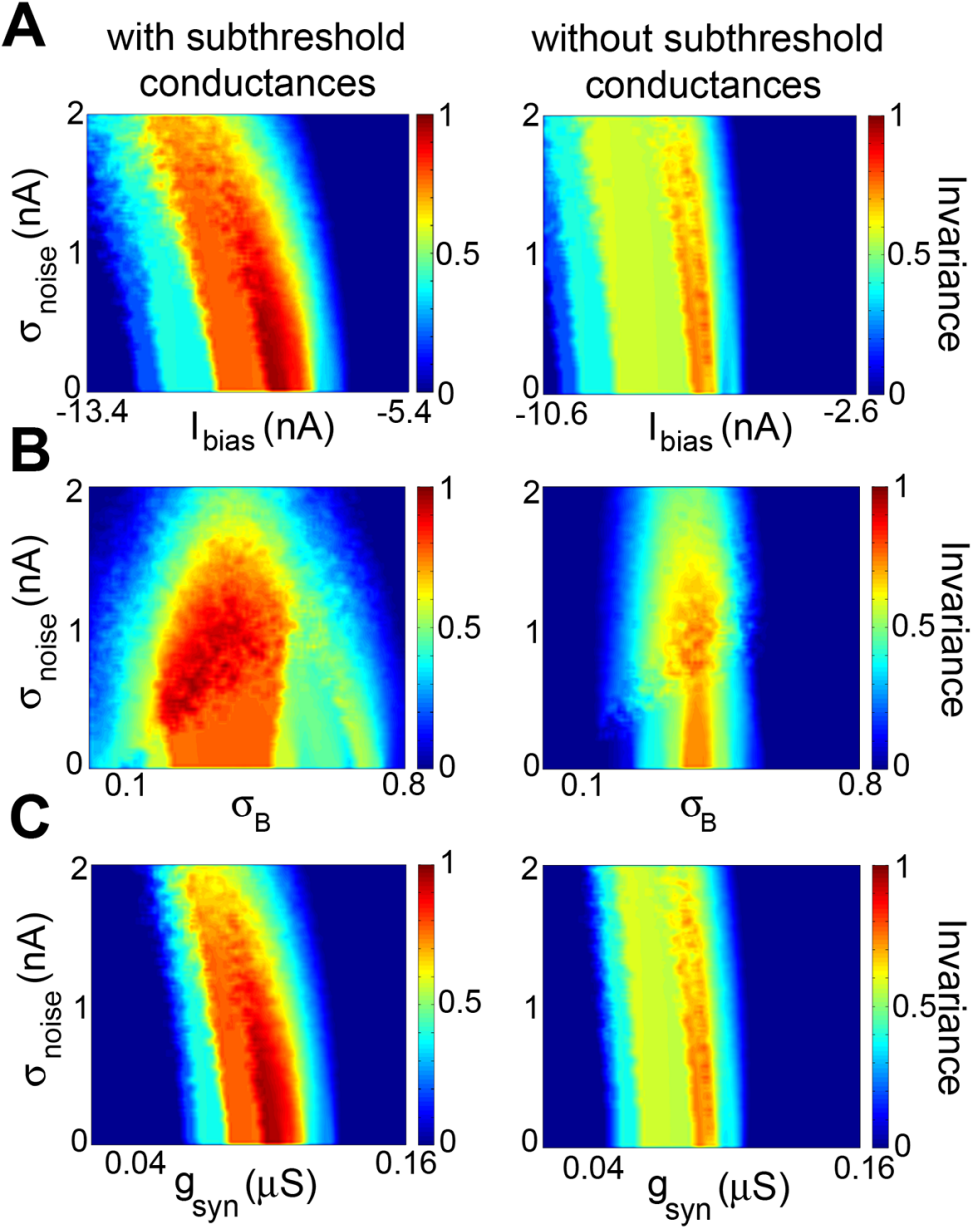
Subthreshold membrane conductances increase the set of parameter values for which feature invariance is observed when varying noise intensity. **A)** Invariance as a function of *I*_*bias*_ and *σ*_*noise*_ with (left) and without (right) the subthreshold membrane conductances *g*_*T*_ and *g*_*h*_. Robustness was 24% (left) vs. 4% (right). **B)** Invariance as a function of *σ*_*B*_ and *σ*_*noise*_ with (left) and without (right) the subthreshold membrane conductances *g*_*T*_ and *g*_*h*_. Robustness was 24% (left) vs. 3.6% (right). **C)** Invariance as a function of *g*_*syn*_ and *σ*_*noise*_ with (left) and without (right) the subthreshold membrane conductances *g*_*T*_ and *g*_*h*_. Robustness was 16.8% (left) vs. 3.6% (right). For the panels on the left, we had *g*_*T*_ = 0.03 μS and *g*_*h*_ = 2.1 μS whereas for those on the right, we had *g*_*T*_ = *g*_*h*_ = 0 μS. Other parameter values were *g*_*syn*_ = 0.102, 0.129 μS; *I*_*bias*_ = –9.39, –6.57 nA; and *σ*_*B*_ = 0.42, 0.41 for the panels on the left and right columns, respectively.

We next investigated why different combinations of parameter values all gave rise to feature invariant responses to natural electrocommunication stimuli. To do so, we systematically varied model parameters. Specifically, we varied the bias current *I*_*bias*_, the T-type calcium conductance *g*_*T*_, the h-type conductance *g*_*h*_, the maximum synaptic conductance *g*_*syn*_, the noise intensity *σ*_*noise*_, and the fraction of ON-type input *σ*_*B*_. We observed negatively sloped bands when varying any two parameters except *σ*_*B*_ within this set (Fig 6A and 6B and 6C as well as the left panels of Figs 7A and 8A and 8C), indicating that increases/decreases in one parameter could be compensated for by decreases/increases in the other parameter. In contrast, we did not observe such negatively sloped bands when varying both *σ*_*B*_ and any of *I*_*bias*_, *g*_*T*_, *g*_*h*_, *g*_*syn*_, or *σ*_*noise*_ (Fig 6D and left panels of Figs 7B and 7C and 8B), indicating that proper tuning of *σ*_*B*_, which gives the relative balance of ON vs. OFF-type input received by the model neuron is necessary to achieve feature invariance as a change in this parameter cannot be compensated for by changing other parameters. We note that, by definition, parameter regions with high feature invariance correspond to regions with high CSI and low VPD values (S1 and S2 Figs). The implications of these findings are discussed below.

### Subthreshold membrane conductances increase regions in parameter space for which feature invariance is observed

While our modeling results have shown that the subthreshold membrane conductances *g*_*h*_ and *g*_*T*_ were not necessary to observe feature invariance, we nevertheless investigated whether these might increase the regions in parameter space for which feature invariance was observed. To do so, we compared the robustness of feature invariance to varying model parameters as quantified by the % of pixels for which we had FI ≥ 0.7 when varying parameters pairwise in models with and without subthreshold membrane conductances. Figs 7 and 8 show the FI values obtained when varying parameters pairwise in model neurons with (left panels) and without (right panels) subthreshold conductances. It is seen that high FI values were obtained for larger regions in parameter space with subthreshold conductances (Figs 7 and 8, compare left and right panels). On average, the robustness with subthreshold conductances was higher (∼16%) than without (∼3%), indicating that the subthreshold membrane conductances *g*_*h*_ and *g*_*T*_, while not necessary to obtain feature invariant responses, do significantly increase the regions in parameter space for which feature invariance is observed.

### Testing the model’s predictions

Our model made important predictions that: 1) a spiking nonlinearity was sufficient to give rise to feature invariance and that 2) maximum feature invariance was obtained when our model neuron received inputs from both ON and OFF-type sources. To test 1), consider that, if a spiking nonlinearity is sufficient to give rise to feature invariance, which first requires that the neural response be selective to the chirp stimulus, then we expect that the membrane potential response would display less selectivity than the spiking response. We tested this hypothesis by comparing CSI values obtained from the membrane potential to those obtained from spikes across chirp stimuli for two neurons that were recorded from intracellularly. Confirming our hypothesis, we found that the CSI values obtained from the membrane potential (0.40±0.06) were significantly less than that obtained from the spiking responses (0.85±0.06) (p = 0.002, signrank test, N = 10).

**Fig 9:**
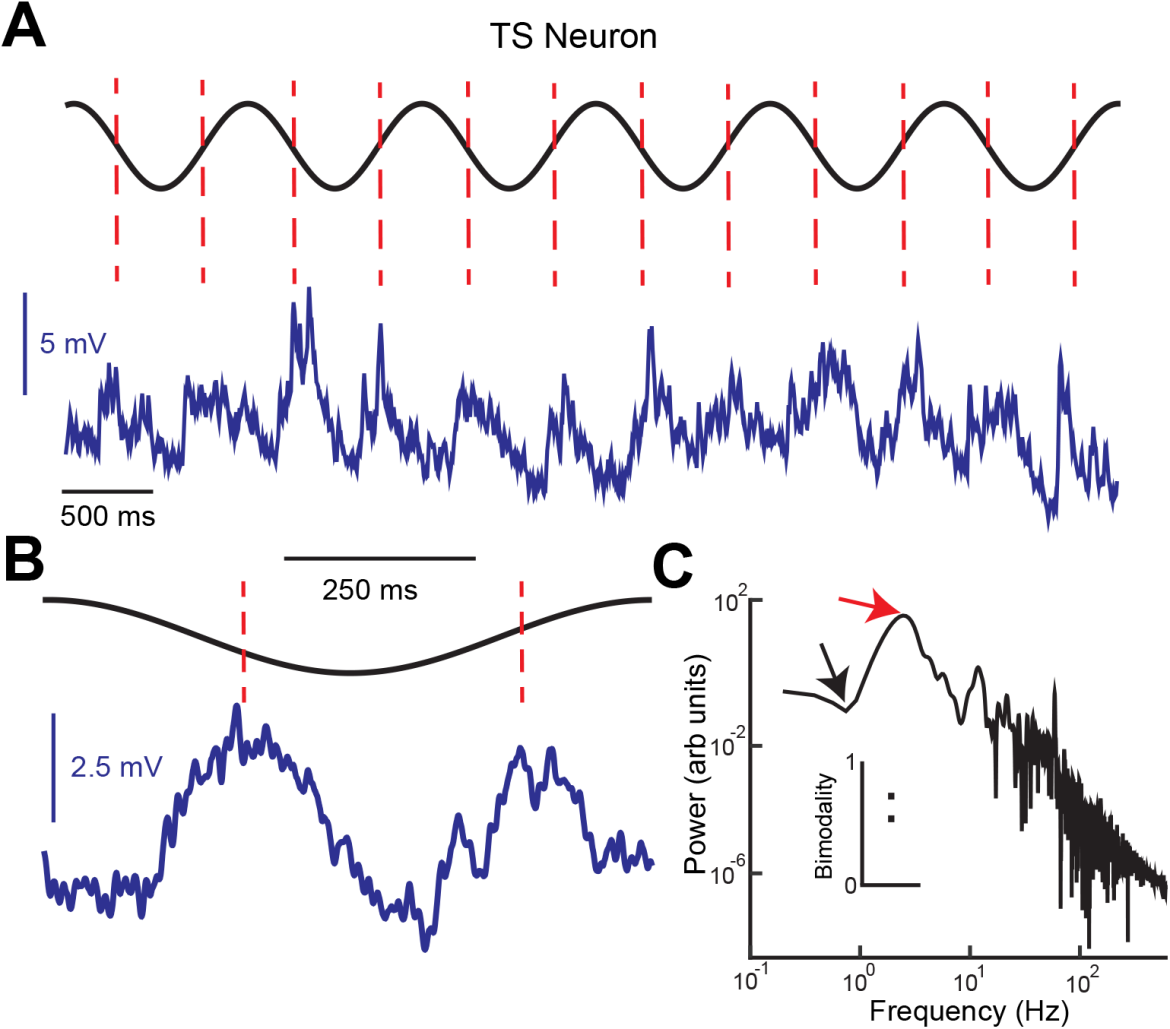
Verifying the model’s prediction. **A)** Membrane potential response (blue) of an example feature invariant TS neuron to sinusoidal stimulation (black). Membrane depolarizations were observed during both the rising and the falling phases of the stimulus (red dashed lines). **B)** The average membrane potential response (blue) during one stimulus cycle (black) was clearly bimodal and displayed two peaks that were approximately 180° out of phase (red dashed lines). **C)** The membrane potential power spectrum displayed most power (red arrow) at frequencies higher than that of the sinusoidal stimulation (black arrow). Inset: Bimodality index computed from 2 feature invariant TS neurons that were recorded from intracellularly.

To test 2), we investigated the membrane potential responses of feature invariant TS neurons to sinusoidal input. We note that ON and OFF-type ELL pyramidal cells respond only during the rising and falling phases of such stimuli, respectively [36, 37]. Thus, if feature invariant TS neurons receive excitatory input from both ON and OFF-type ELL pyramidal cells, then we would expect to see membrane potential depolarizations during both the rising and falling phases of the sinusoidal stimulus. Fig 9A shows the membrane potential response of an example TS neuron to sinusoidal stimulation. Confirming our hypothesis, we observed depolarizations during both the rising and falling phases of the stimulus (Fig 9A, dashed red lines). The average membrane potential response to one stimulus cycle (Fig 9B) was clearly bimodal. This was confirmed by computing the power spectral density of the membrane potential that showed maximum power at twice the stimulus frequency (Fig 9C, red arrow) and much less power at the stimulus frequency (Fig 9C, black arrow). We computed a bimodality index whose value is 1 if the neuron responds equally at two phases π radians apart and zero if the neuron only responds at one phase (see Methods). We found values of 0.67 and 0.52 for both TS neurons (Fig 9C, inset). The implications of these results are discussed below.

## Discussion

### Summary of results

We investigated the coding of natural electrocommunication stimuli in both hindbrain and midbrain electrosensory neurons. Our characterization of natural electrocommunication stimuli revealed strong heterogeneities in waveforms that gave rise to differential response patterns in hindbrain pyramidal neurons. Surprisingly, we found that some TS neurons displayed feature invariant responses to natural electrocommunication stimuli through nonlinear integration of balanced hindbrain ELL neuron afferent input. In order to understand the underlying mechanisms, we built a model based on the Hodgkin-Huxley formalism. Systematically varying model parameters and finding parameter values giving rise to the highest levels of feature invariance through an evolutionary algorithm revealed that very different combinations of parameter values could give rise to approximately similar degrees of feature invariance. This was because the effects of changing a given parameter could be compensated for by changing another parameter. This compensation was seen for all parameters tested with the notable exception of the balance between ON and OFF-type inputs. Although a spiking nonlinearity was sufficient to observe feature invariance, our results nevertheless showed that subthreshold membrane conductances enhanced the robustness of feature invariance overall. We then verified our modeling predictions experimentally and found that: 1) the membrane potential response was less selective to chirps than the spiking response; 2) feature invariant TS neurons responded with membrane potential depolarizations during both the rising and the falling phase of sinusoidal stimuli suggesting that they do indeed receive input from both ON and OFF-type ELL pyramidal neurons.

### Multiple combinations of model parameters lead to feature invariant responses

Our model made the important novel prediction that multiple combinations of parameter values can give rise to feature invariant responses to natural electrocommunication stimuli. The occurrence of similar neural network output despite considerable differences in underlying cellular properties has been previously observed in mathematical models [41–46]. However, such phenomena have mostly been observed experimentally in invertebrate model systems where a given neuron type can be reliably identified across individuals [47–51]. The invariance in output pattern is thought to promote robust function despite perturbations or variability during development [49, 52, 53] by permitting homeostasis through compensatory mechanisms [54–56] as well as genetic alterations [49, 57]. Our results have shown that feature invariant responses to natural electrocommunication stimuli could be obtained in a realistic model through multiple combinations of parameter values. This was because changes in some parameters could be compensated for through changes in others, thereby leading to correlations between parameters as observed elsewhere [58]. Previous studies have suggested that such optimization can be achieved through coupled control of ion channels [48, 53, 58], which could be applicable for h- and T-type currents in this case. Such studies are needed to uncover whether the electrosensory system uses most if not all of the solutions available to give rise to feature invariance. In general experimental studies have found the variability in neuronal and circuit properties to be less than that found in mathematical models: this is likely due to an incomplete understanding of the more complex biological constraints imposed on the molecular, cellular, and network levels [51, 52, 59]. Interestingly, we found that not all changes in parameter values could be compensated for as the best feature invariant responses were observed when the model TS neuron received nearly balanced inputs from ON and OFF-type ELL pyramidal neurons, which was confirmed experimentally. This suggests that these TS neurons correspond to the “ON-OFF” (or type 3) neurons that have been previously observed in TS [60, 61] and that were found to respond selectively to the second order features of electrosensory stimuli [40]. The fact that the neurons responding selectively to the second order features of electrosensory stimuli considered previously tended to spike in response to sinusoidal stimuli [40] whereas the neurons displaying selective but invariant responses to chirps considered here did not suggest that these are not the same neuron type. Since only a small percentage (∼5%) of TS neurons in our dataset displayed selective and invariant responses to chirps, we hypothesize that these must correspond to 1 of the 50 previously anatomically identified cell types within TS [62]. An experimental verification of these predictions is at best challenging as it would require identification of which neural class(es) in TS show selective and invariant responses to chirps as well as selective responses to second order features of electrosensory stimuli. Such experiments should also directly verify whether these neurons do indeed receive inputs from both ON and OFF-type ELL pyramidal neurons through direct stimulation of afferent synaptic connections *in vitro*. These studies are beyond the scope of this paper. Moreover, while previous studies have found that TS neurons receive large amounts of neuromodulatory inputs [63, 64], the effects of these on sensory processing have only been studied in hindbrain pyramidal neurons [34, 65–67]. Thus, further studies are needed to understand how neuromodulators affect sensory processing within TS and whether the feature invariant responses seen in this study are robust to such neuromodulators as seen elsewhere [68].

We also note that previous anatomical studies have found that TS neurons receive inhibitory input exclusively from other neurons located within TS [62]. While our modeling results show that such inhibition is not necessary in order to observe selective but feature invariant responses to chirps, it is conceivable that such inhibition could be used to enhance response selectivity as well as similarity. Further studies are needed to understand the role played by inhibition on sensory processing by TS neurons.

Finally, we note that future studies should test whether TS neurons display feature invariant responses to more chirp waveforms. In particular, previous studies have reported that small chirps can occur on top of high frequency beats [24, 29]. The fact that TS neurons responded similarly to both big chirps occurring on top of high frequency beats and small chirps occurring on top of low frequency beats would suggest that they would respond similarly to small chirps occurring on top of high frequency beats but further studies are required to test this prediction.

### Feature invariant responses to natural electrocommunication stimuli: Consequences on perception/behavior

While previous studies have reported that both peripheral electroreceptor [27–29] as well hindbrain pyramidal [30–32, 69] neurons can respond to natural electrocommunication stimuli, these have all shown differential responses to different chirp waveforms. In contrast, behavioral studies have shown that weakly electric fish display robust and similar behavioral responses to small chirps despite such heterogeneous waveforms [23, 24, 70], thereby suggesting that the animal perceives these waveforms as similar. If this hypothesis is true, then our results showing that some TS neurons respond in a feature invariant manner would provide a neural correlate of such invariant behavioral responses implying that perception of natural electrocommunication stimuli is largely independent of their characteristics. However, previous studies have shown large heterogeneities in the responses of TS neurons to stimuli: some neurons respond to moving objects in a directionally selective manner [39, 71, 72], others to second order stimulus features [40], and others to natural electrocommunication stimuli [32]. Such diversity in responses is likely to be a signature of several different parallel processing pathways for natural electrosensory stimuli. The feature invariant responses of some TS neurons to natural electrocommunication stimuli are likely to serve as a reliable detection signal for their occurrence in time [32]. Such a signal would be advantageous as small and big chirp stimuli respectively occur during aggressive and courtship behavior [23, 24, 26], which might help the animal better prepare for each context.

It is nevertheless possible that previously described other TS neurons that respond selectively but differentially to different chirp waveforms convey information about chirp attributes in parallel [32]. This is an attractive hypothesis as these TS neurons tended to respond either to small or to big chirps, which would provide a neural correlate of behavioral results suggesting that the animal can actually distinguish between small and big chirp waveforms, which is consistent with the fact that big chirps instead constitute an attractive signal for a potential mate (see [26] for review). Behavioral studies in which the behavioral responses to different small and big chirp waveforms are explicitly considered and compared are needed to test whether the animal can actually distinguish between different waveforms and to test whether and, if so, how perception depends on natural electrocommunication stimulus attributes.

We furthermore note that previous studies have found that electrosensory pyramidal neurons display large heterogeneities and can be classified into different classes that are associated with differential expression of ionic conductances [73, 74], amount of descending input [37, 75], and dendritic morphology [76]. Anatomical studies have shown that the electrosensory lateral line lobe is organized into columns each containing one member from each pyramidal cell class. While all classes project to the midbrain TS [75], the specific pattern of innervation is not known. Further studies are needed to uncover this pattern.

### Implications for other systems

The emergence of feature invariant neuronal responses appears to be a ubiquitous strategy for sensory processing across species and systems. Indeed, such neurons have been observed in both the visual [6, 12, 14, 77, 78] and auditory [9–11, 79] pathways. In general, previous studies have noted an increase in both response selectivity and invariance as information propagates to higher brain regions [10, 12, 14]. Thus, feature invariance appears to be linked to sparse coding [1]. Moreover, it is generally agreed that feature invariance develops in stages with neurons in higher brain areas displaying progressively more invariant responses [10, 12, 14]. Hence, our results showing that some TS neurons display feature invariant responses to natural electrocommunication stimuli, together with previous results showing the emergence of sparse coding in TS [32], are consistent with those obtained elsewhere. In particular, we found that nonlinear integration of balanced input from ON and OFF-type neurons was sufficient to give rise to feature invariant responses. This mechanism is likely to be applicable to other systems: this is because neurons in more peripheral brain areas also tend to respond to identity preserving transformations of a stimulus through different patterns of excitation and inhibition [79]. Further, ON and OFF-type neurons have been observed in the more peripheral brain areas (e.g. retina) of other systems [80, 81] and studies have shown that higher order neurons likely receive mixed input from both ON and OFF-type neurons [82, 83]. Additionally, the membrane conductances used in our model are generic and found ubiquitously in the central nervous system [84–86]. These observations, together with many anatomical and physiological similarities between the electrosensory and other systems (see [17–19] for review), suggest that the results obtained in this study are applicable to other systems.

### Conclusion

We have provided the first experimental evidence that some midbrain electrosensory neurons respond to heterogeneous natural electrocommunication stimuli selectively but in an invariant manner. We have further shown that a simple model receiving input from ON and OFF-type hindbrain neurons that is consistent with known anatomy could reproduce experimental findings for multiple parameter combinations. While a spiking nonlinearity was sufficient to give rise to feature invariance in our model, the addition of subthreshold membrane conductances increased the set of parameter values leading to such invariance. It is likely that the mechanisms giving rise to feature invariance in the electrosensory system will also be found elsewhere.

## Methods

### Ethics statement

McGill University’s animal care committee approved all procedures. McGill University holds a certificate of ‘Good Animal Practice’ from the Canadian Council on Animal Care and is also certified by the US National Institutes of Health Public Health Service under the 'Policy on Humane Care and Use of Laboratory Animals' with Assurance number A-5006-01.

### Animals and surgery

The weakly electric fish *Apteronotus leptorhynchus* was used exclusively in this study. Fish were acquired from tropical fish suppliers, acclimated to the laboratory as per published guidelines [87, 88]. Surgical procedures were explained in detail previously [32, 34, 39, 40, 89, 90]. The animal was immobilized with 0.1–0.5 mg injection of tubocurarine (Sigma) intramuscularly. The fish was then transferred to a recording tank and respirated via a mouth tube with flow rate of 10 mL/min. We then glued a metal post rostral to the exposed area of the skull after topical application of lidocaine (2%) to ensure stability during recording. We then drilled a small hole of ∼2 mm^2^ over the cerebellum and the ELL area in the case of ELL recordings [34, 91–94], and over the midbrain optic tectum in the case of TS recordings [32, 38–40].

### Behavior

Fish were restrained by placing them in a “chirp chamber” as previously described [34]. Chirps were identified as increases in the animal’s own EOD frequency that exceeded 30 Hz and were segregated into small (type II) and big (type I) as done previously [33]. We recorded chirp responses to sinusoidal waveforms mimicking another fish’s EOD whose frequency was set 10 and 80 Hz above the animal’s own EOD frequency for type II and type I chirps, respectively. The fish’s EOD was recorded and the instantaneous EOD frequency was computed from the inverse of the timing difference between successive zero crossings. The chirp duration was defined as the full width at half-maximum of the frequency increase. The time of occurrence of the chirp was defined as the time at which the EOD frequency is maximal. The beat phase at which the chirp occurred was obtained by expressing the time at which the chirp occurred at relative to the nearest time occurrence of a local maximum of the beat in the past, dividing by the beat period, and multiplying the result by 360°. We computed a similarity metric to capture the variability for small and big chirps that was defined by:

SM=1−RMSE/σ,

where RMSE is the root-mean squared error between two chirp waveforms computed as:

$$RMSE=\sqrt{\left\langle {\left[S_{i}-\langle S_{i}\rangle -S_{j}+\left\langle S_{j}\right\rangle \right]}^{2}\right\rangle }$$

and *σ* is the maximum error given by:

$$\sigma =\max\left[\frac{\max\left(S_{i}\right)-\min\left(S_{i}\right)}{\sqrt{2}},\frac{\max\left(S_{j}\right)-\min\left(S_{j}\right)}{\sqrt{2}}\right]$$

Here <…> denotes the average over time which was computed over a time window of 37.5 ms centered on the chirp onset. Density plots were constructed using a binwidth of 0.55 ms for duration, 14 degrees for the phase distribution and a binwidth of 0.038 for the similarity metric. The number of events occurring per bin were counted and normalized to the maximum number of events found. To assess whether the phase at which a chirp (small chirp or big chirp) occurred was homogeneously distributed, we generated 1000 surrogate phase values. These were obtained by randomly permuting a set of phase values between 0 and 2π with the same number of elements as the actual dataset for small and big chirps. The confidence interval was set to be 3 times the standard deviation obtained from the surrogate phase values.

### Recordings

Extracellular recordings were made from pyramidal cells within the lateral segment of the ELL because these are most sensitive to the stimuli used in this study [30, 31] and from TS neurons using metal filled micropipettes [95] as described previously [91]. Intracellular recordings from TS neurons were made using patch pipettes as described previously [71, 72, 96]. The recorded signals were sampled at 10 kHz and were digitized by a Power1401 with Spike2 software. ON and OFF-type pyramidal cells can easily be distinguished based on their responses to sinusoidal stimuli as their responses are then in and out of phase, respectively [36].

### Stimulation

We note that the electric organ of *A*. *leptorhynchus* is neurogenic and is thus not affected by injection of curare-like drugs. All stimuli consisted of amplitude modulations (AMs) of the animal’s own EOD. They were produced by first generating a train of sinusoidal waveforms that were triggered by the zero crossing of each EOD cycle with a frequency slightly greater than the fish’s own EOD frequency. The train thus remains synchronized to the animal’s EOD and will either add or subtract depending on its polarity. The modulation waveform (i.e. the stimulus) is then multiplied (MT3 multiplier, Tucker Davis Technologies) with the train and the resulting signal was applied to the experimental tank after being isolated from ground (A395 linear stimulus isolator, World Precision Instruments) via two chloridized silver wire electrodes located ~15 cm on each side of the animal [37]. Stimulus intensities were similar to those used in previous studies [34, 91]. Chirp stimuli were generated as previously described [32] and were each presented at least 20 times to each neuron.

### Analysis

All analysis was performed using custom-built routines in Matlab (The Mathworks, Natick, MA). Action potential times were defined as the times at which the signal crossed a suitably chosen threshold value. From the spike time sequence we created a binary sequence X(t) with binwidth dt = 0.5 ms and set the content of each bin to equal the number of spikes the time of which fell within that bin. Peri-stimulus time histograms (PSTHs) were obtained by averaging the neural responses across repeated presentations of a given stimulus with binwidth *Δt* = 0.1 ms and were smoothed with a 10.8 ms long boxcar filter for the small chirps and 5 ms for the big chirp.

To quantify the selectivity of a given neuron for the chirp stimulus, the chirp selectivity index (CSI) was computed as follows:

$$CSI=\frac{R_{C}-R_{B}}{R_{C}+R_{B}}$$

where *R*_*C*_ and *R*_*B*_ represent the maximum firing rates of the PSTH during the chirp and beat, respectively, similar to what was done previously [32]. The window used to define the chirp time was 100 ms in length, starting at the onset of the chirp in the stimulus. The CSI ranges between -1 and 1, representing perfect selectivity for the beat at -1 and the chirp at 1. To measure the selectivity of a model across multiple chirp stimuli, the average CSI was used:

$$CSI_{avg}=\frac{1}{N}\overset{N}{\underset{i=1}{\sum }}CSI_{i}$$

where N is the number of chirp stimuli tested and *CSI*_*i*_ is the *CSI* to chirp stimuli *i*. We also computed the CSI from the membrane potential minus its minimum value in the same way as described above.

We first computed the average membrane potential response to sinusoidal stimulation. The bimodality index was computed from the average membrane potential response minus its minimum value in the same way as described previously [40]. First, we performed a circular permutation such that the maximum signal value is now located at 0. The bimodality index was then obtained by dividing the signal value at half the stimulus period by the signal value at 0.

The invariance of a neuronal response across different stimuli was quantified by comparing the similarity of the spike trains across chirp types and trials. The Victor-Purpura distance (*VPD*), a metric-space measure of the distance between two spike trains, was used to quantify this [35]. Briefly, the *VPD* computes the total cost of transforming one spike train into another via an optimal series of elementary operations and used *q =* 100 s^−1^ as done previously [32]. Thus, the feature invariance of a neuron was computed by the average *VPD* across all pairs of trials as follows:

$$VPD_{avg}=\frac{1}{M}\overset{N}{\underset{i=1}{\sum }}\overset{N}{\underset{j=i}{\sum }}\overset{N_{T}}{\underset{k=1}{\sum }}\overset{N_{T}}{\underset{l=u_{ij}}{\sum }}VPD\left(\;C_{i}\left(k\right),C_{j}\left(l\right)\;\right)$$

where *N*_*T*_ is the number of trials for each chirp stimulus, *M* is the number of combinations of pairs of trials (i.e. the number of times the *VPD* is computed), *u*_*ij*_ is 1 if *i* ≠ *j* and is *k* + 1 otherwise, and *VPD*(*C*_*i*_(*k*),*C*_*j*_(*l*)) represents the *VPD* between the spike trains in response to the *k* ^*th*^ presentation of chirp *i* and the *l* ^*th*^ presentation of chirp *j*, respectively.

For computing the invariance of neural responses of population averages, individual spike trains were not accessible for calculating the *VPD*. Therefore, the root-mean squared error (RMSE) between the PSTHs of the responses of the populations to the various chirps was computed instead. Average PSTHs were computed by averaging the PSTHs across a given population of cells for each chirp type, and these were used to compute the average pairwise RMSE between all pairs of PSTHs as follows. First, in order to prevent small differences in timing from biasing the result (because an identical response shifted slightly in time will have a high RMSE), a cross-correlogram was computed between the two PSTHs, followed by a circular shift of one PSTH by the time shift at the maximum value of the cross-correlogram (i.e. leading to maximum overlap between the PSTHs). These PSTHs were then used to compute the final average RMSE as:

$$RMSE_{avg}=\frac{1}{P}\overset{N}{\underset{i=1}{\sum }}\overset{N}{\underset{j=i}{\sum }}\sqrt{\frac{1}{L}\sum_{k=1}^{L}{\left(\;PSTH_{i}\left(k\;\Delta t\right)-PSTH_{j}\left(k\;\Delta t\right)\;\right)}^{2}}$$

where *P* is the number of pairs of PSTHs considered, *L* is the number of bins for the PSTH and Δt is the binwidth.

The measures for selectivity and spike train invariance were used to compute a feature invariance (FI) score as follows:

$$FI=H_{0}\left(CSI_{avg}-\alpha \;VPD_{avg}\right)$$

where *H*_*0*_ is the Heaviside step function, and *α* >0 is a constant. We note that the choice of α>0 is arbitrary and does not affect the qualitative nature of our results as long as the value chosen is not too large. We chose α = 0.01 in order to emphasize the fact that the responses of some TS neurons to different chirp waveforms were far more similar and selective than those of ELL neurons. We only included TS neurons for which we obtained FI>0.2. Importantly, we obtained FI<0.2 for all ELL neurons in our dataset.

For computing FI scores of population averages via their PSTHs, an FI score based on the RMSE was instead used:

$$FI_{RMSE}=H_{0}\left(CSI_{avg}-\gamma RMSE_{avg}\right)$$

where *γ* = 0.0041 is a constant whose value was chosen by requiring that *FI = FI*_*RMSE*_ for a representative, feature invariant TS neuron. We also compared *VPD*_*avg*_ and *RMSE*_avg_ computed from single ELL and TS neuron spike train and PSTH responses, respectively. Overall, we found a strong positive correlation between both quantities (R = 0.90, N = 54, p<<10^−3^), indicating that using either measure will not affect our results qualitatively. We furthermore found similar values of *FI* and *FI*_*RMSE*_ for ELL ON-cells (0.000±0.000 vs. 0.0055±0.0036), OFF-cells (0.004±0.004 vs. 0.0232±0.0180), and TS neurons (0.54±0.09 vs. 0.577±0.082).

### Statistics

Statistical significance was assessed through one-way analysis of variance (ANOVA) with the Tukey-Kramer method of correcting for multiple comparisons at the p = 0.05 level. Values are reported as mean ± standard error throughout the text.

### Model

Our model is based on the Hodgkin-Huxley formalism and considers a single TS neuron receiving excitatory synaptic input from ON and OFF-type ELL pyramidal cell populations based on known anatomical data [62].

We obtained the synaptic input by pooling the recorded activities of ON and OFF-type pyramidal cells in response to the chirp stimuli used in this study. The population-averaged PSTH was then converted to a time varying conductance as follows:

$$\begin{array}{l} g_{ON}\left(t\right)=\zeta \;g_{\max}\left(PSTH_{E}\left(t\right)*\left[\frac{t}{\tau_{syn}}\exp\left(1-\frac{t}{\tau_{syn}}\right)H_{0}\left[t\right]\right]\right)\\ g_{OFF}\left(t\right)=\zeta \;g_{\max}\left(PSTH_{I}\left(t\right)*\left[\frac{t}{\tau_{syn}}\exp\left(1-\frac{t}{\tau_{syn}}\right)H_{0}\left[t\right]\right]\right) \end{array}$$

where “*” is the convolution operator and *PSTH*_*E*_, *PSTH*_*I*_ are the population-averaged PSTHs for ON and OFF-type pyramidal cells, respectively. We used *g*_max_ = 0.13 μS, *ζ* = 0.0005, and *τ*_*syn*_ = 20 ms. The time varying synaptic current applied to the model TS neuron was then equal to:

$$\begin{array}{l} I_{syn}(t)=-2\;W_{s}\left(\sigma_{B}g_{ON}(t)+\left(1-\sigma_{B}\right)g_{OFF}(t)\right)\left(V-E_{syn}\right)\\ \;=-2\;g_{syn}\left(\sigma_{B}g_{ON}(t)/g_{\max}+\left(1-\sigma_{B}\right)g_{OFF}(t)/g_{\max}\right)\left(V-E_{syn}\right) \end{array}$$

where *W*_*s*_ is a constant that was varied systematically, the parameter *σ*_*B*_ controls the relative proportion of input from ON-type pyramidal cells, which is important as previous studies have shown that this proportion can vary substantially across TS neurons [40], *E*_*syn*_ = 0 mV is the reversal potential of the excitatory synapse. Note that we report the value of *g*_*syn*_ = *W*_*s*_ *g*_*max*_ in the figures.

TS neurons were modeled with the Hodgkin-Huxley formalism [97], via the following system of stochastic differential equations:

$$\begin{array}{l} \frac{dV}{dt}=\frac{1}{C}\left(I_{Na}+I_{KDR}+I_{h}+I_{T}+I_{leak}+I_{syn}+I_{bias}+\sigma_{noise}\;\xi (t)\right)\\ \frac{d\eta }{dt}=\Phi \frac{\eta_{\infty }(V)-\eta }{\tau_{\eta }}\\ \frac{dh}{dt}=\frac{h_{\infty }(V)-h}{\tau_{h}}\\ \frac{dn}{dt}=\frac{n_{\infty }(V)-n}{\tau_{n}} \end{array}$$

where *C* = 1 μF is the cell membrane capacitance, *V* is the transmembrane voltage, *I*_*Na*_ is the spiking sodium current, *I*_*KDR*_ is the delayed rectifier potassium current, *I*_*h*_ is the hyperpolarization activated current mediated by HCN channels, *I*_*T*_ is the low-threshold T-type calcium channel current, *I*_*syn*_ is the synaptic current from the ELL defined above, *I*_*leak*_ is the leak current, *I*_*bias*_ is a constant bias current, and *η*, *h*, and *n* represent the voltage-dependent channel activation variables with *Φ* = 2 and *τ*_*η*_ *=* 30 ms. *ξ(t)* is a time-varying stochastic Gaussian white noise process with mean zero and standard deviation 0.8. We used *σ*_*noise*_ = 1 nA unless otherwise noted. The equations governing T-type calcium, sodium, and delayed rectifier potassium channel activation/inactivation were as follows:

$$\begin{array}{l} m_{\infty }(V)=\frac{\alpha_{m}(V)}{\alpha_{m}(V)+\beta_{m}(V)}\\ \alpha_{m}(V)=\frac{0.1\left(V+40.7\right)}{1-\exp\left(-0.1\left(V+40.7\right)\right)}\\ \beta_{m}(V)=4\exp\left(-0.05\left(V+49.7\right)\right)\\ \eta_{\infty }(V)=\frac{1}{0.5+\sqrt{0.25+\exp\left(\frac{V+82}{6.3}\right)}}\\ s_{\infty }(V)=\frac{1}{1+\exp\left(-\frac{V+63}{7.8}\right)}\\ n_{\infty }(V)=\frac{\alpha_{n}(V)}{\alpha_{n}(V)+\beta_{n}(V)}\\ \alpha_{n}(V)=\frac{0.01\left(V+40.7\right)}{1-\exp\left(-0.1\left(V+40.7\right)\right)}\\ \beta_{n}(V)=0.125\exp\left(-0.0125\left(V+50.7\right)\right)\\ \tau_{n}(V)=\frac{0.05}{\alpha_{n}(V)+\beta_{n}(V)}\\ h_{\infty }(V)=\frac{1}{1+\exp\left(0.151\left(V+73\right)\right)}\\ \tau_{h}=\frac{\exp\left(0.033\left(V+75\right)\right)}{0.011\left[1+\exp\left(0.083\left(V+75\right)\right)\right]} \end{array}$$

and the ionic currents are given by:

$$\begin{array}{l} I_{Na}=-g_{Na}\;m_{\infty }^{3}(V)\left(0.85-n\right)\left(V-E_{Na}\right)\\ I_{KDR}=-g_{k}\;n^{4}\;\left(V-E_{K}\right)\\ I_{h}=-g_{h}\;h\left(V-E_{h}\right)\\ I_{T}=-g_{T}\;s_{\infty }^{3}(V)\eta \;(V-E_{Ca})\\ I_{leak}=-g_{leak}\left(V-E_{leak}\right) \end{array}$$

where the reversal potentials of the ionic conductances are given by: *E*_*Na*_ = 60 mV, *E*_*K*_ = -85 mV, *E*_*leak*_ = -65 mV, *E*_*h*_ = -30 mV, *E*_*Ca*_ = 120 mV. The maximal conductances, unless otherwise noted, are given by *g*_*Na*_ = 30 μS, *g*_*K*_ = 10 μS, *g*_*leak*_ = 0.18 μS, *g*_*h*_ = 7 μS, and *g*_*T*_ = 0.32 μS. Parameters for *I*_*h*_ were the same as those used previously [84, 98]. We concentrated on the subthreshold currents *I*_*T*_ and *I*_*h*_ because these can promote spiking following hyperpolarization and have furthermore been shown to be present in midbrain neurons [71, 99].

The above system of equations was numerically simulated with the Euler-Maruyama algorithm [100], using an integration time-step of 0.025 ms. The model TS neuron’s output was analyzed in the same fashion as the experimental data. The robustness of feature invariance to changes in model parameter values was computed as the % of pixels for which we had FI ≥ 0.7.

### Differential evolution search algorithm

We used a differential evolution (DE) algorithm [101, 102] in order to find sets of parameter values for which the model neuron’s output to different input chirp waveforms matched that seen experimentally in TS neurons. Specifically, we implemented a variant of a previously proposed method for global parameter estimation of Hodgkin-Huxley models [102]. DE evolves a set (or population) of parameter vectors (i.e. “individuals”) by minimizing a fitness function *F*_fit_ over a series of iterations (i.e. “generations”). In keeping with the notation used in previous studies [101, 102], we denote $X_{k}^{r}\left(i\right)$ as parameter *i* for individual *r* of generation *k*. First, the population of *K* individuals is randomly initialized with values for each of the *D* parameters, uniformly distributed within the boundary constraints for each. Here, the parameter vector is made up of *σ*_*B*_, *W*_*S*_, *I*_*bias*_, *g*_*h*_, *g*_*T*_ (i.e. D = 5). For each individual at every generation, a new individual is constructed by two operations consisting of "differentiation" and "recombination". In differentiation, the *r* ^*th*^ new parameter vector $X_{k,trial}^{r}$ is built by combining three other individuals $X_{k}^{r_{1}}$, $X_{k}^{r_{2}}$, and $X_{k}^{r_{3}}$, where *r*_*1*_≠*r*_*2*_≠*r*_*3*_:

$$X_{k,trial}^{r}=X_{k}^{r_{1}}+\left(X_{k}^{r_{2}}-X_{k}^{r_{3}}\right)F\;\forall \;r=1,\ldots ,N$$

where the differential weight *F* = 0.5, and the three individuals are chosen based on a probability distribution that is preferentially weighted for more fit (i.e. lower fitness score) individuals:

$$p_{k}^{r_{i}}=\lambda \exp\left(\frac{-F_{fit}\left(X_{k}^{r_{i}}\right)}{\underset{\forall j}{\max}(F_{fit}(X_{k}^{j}))}\right)\;\forall \;r_{i}=1,\ldots ,N$$

where *λ* is a normalization constant such that the sum of probability values is equal to one. Recombination is then performed as follows:

$$X_{mut}^{r}\left(i\right)=\{\begin{array}{l} \;X_{k,trial}^{r}\left(i\right)\;if\;u<CR\\ \;X_{k}^{r}\left(i\right)\;otherwise \end{array}\;\forall \;r=1,\ldots ,N;\;i=1,\ldots ,D$$

where *u* is a random variable generated from a uniform distribution *U*(0,1) and with crossover probability *CR* = 0.9. Selection is finally performed to produce the next generation via:

$$X_{k+1}^{r}=\{\begin{array}{l} \;X_{mut}^{r}\;if\;F_{fit}\left(X_{mut}^{r}\right)\leq F_{fit}\left(X_{k}^{r}\right)\\ \;X_{k}^{r}\;otherwise \end{array}\;\forall \;r=1,\ldots ,N$$

In this study, the fitness function for a given individual was defined as:

$$F_{fit}\left(X_{k}^{r}\right)=\exp\left(-FI_{X_{k}^{r}}\right)$$

where $FI_{X_{k}^{r}}$ is the FI score computed from simulating a model using the parameters encoded in individual $X_{k}^{r}$ on the five chirp stimuli. To encode boundary constraints of the parameter values within physiologically realistic ranges, the “resampling” approach was adopted, based on previous empirical results [103]. Whenever a parameter (gene) value violates its constraints, resampling enforces the boundary conditions by rerunning the differentiation step with three new individuals chosen uniformly randomly.

## Supporting Information

S1 Fig Plots of VPD (top) and CSI (bottom) as a function of: A) *g*_*T*_ and *I*_*bias*_; B) *g*_*T*_ and *σ*_*noise*_; C) *g*_*T*_ and *g*_*syn*_; D) *g*_*T*_ and *σ*_*B*_. Parameter values are the same as in Fig 6.
(TIF)

S2 Fig Plots of VPD (top) and CSI (bottom) as a function of: A) *g*_*h*_ and *g*_*T*_; B) *g*_*h*_ and *I*_*bias*_; C) *g*_*h*_ and *g*_*syn*_; D) *g*_*h*_ and *σ*_*B*_. Parameter values are the same as in Fig 6.
(TIF)
