# Supplementary figures and images for: Electrosensory Midbrain Neurons Display Feature Invariant Responses to Natural Communication Stimuli
Source: PLoS Comput Biol. 2015 Oct 16;11(10):e1004430. doi: 10.1371/journal.pcbi.1004430 (PMC4608831; doi:10.1371/journal.pcbi.1004430)

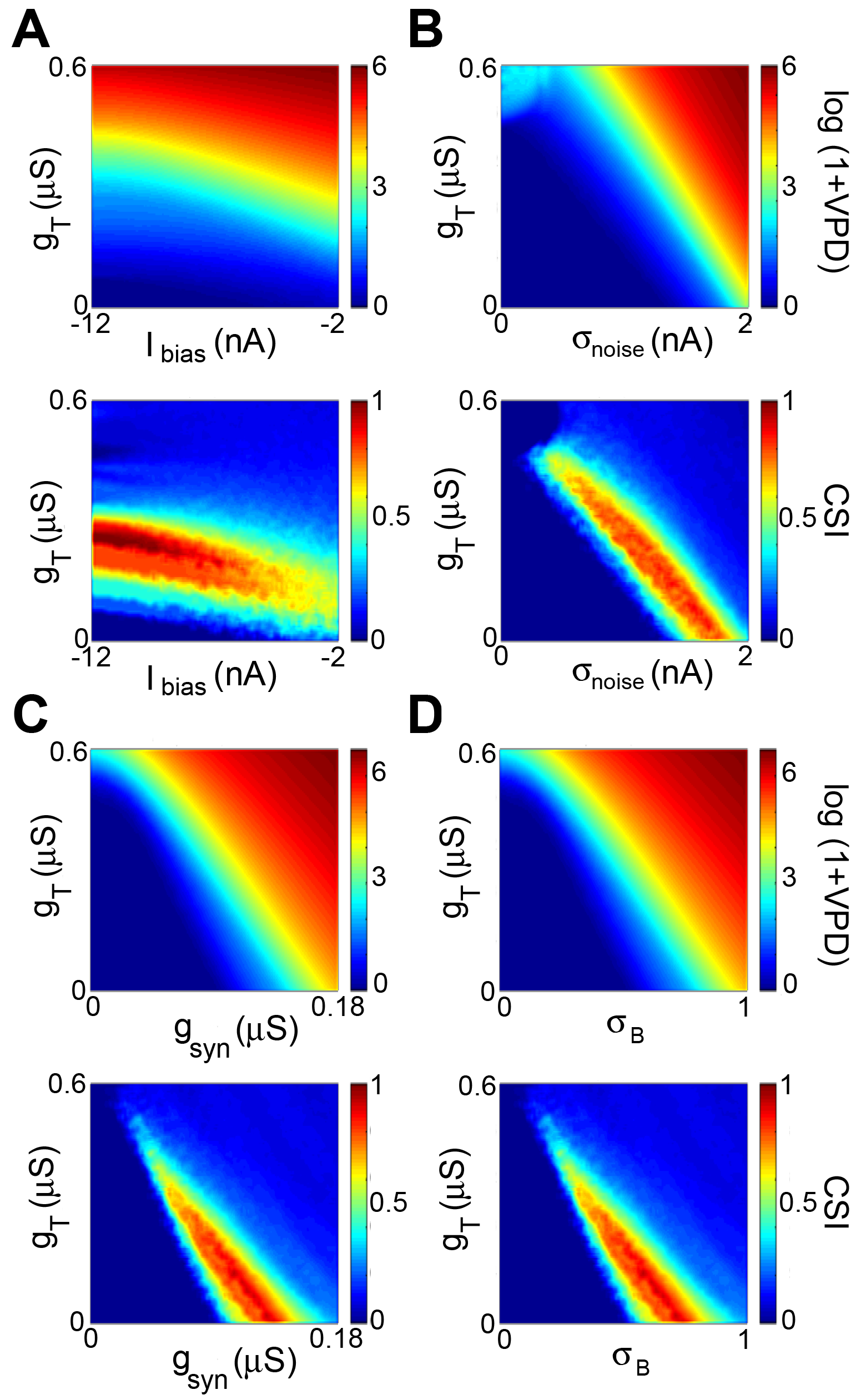

Supplement: S1 Fig — Parameter values are the same as in Fig 6. (TIF) [file pcbi.1004430.s001.tif]

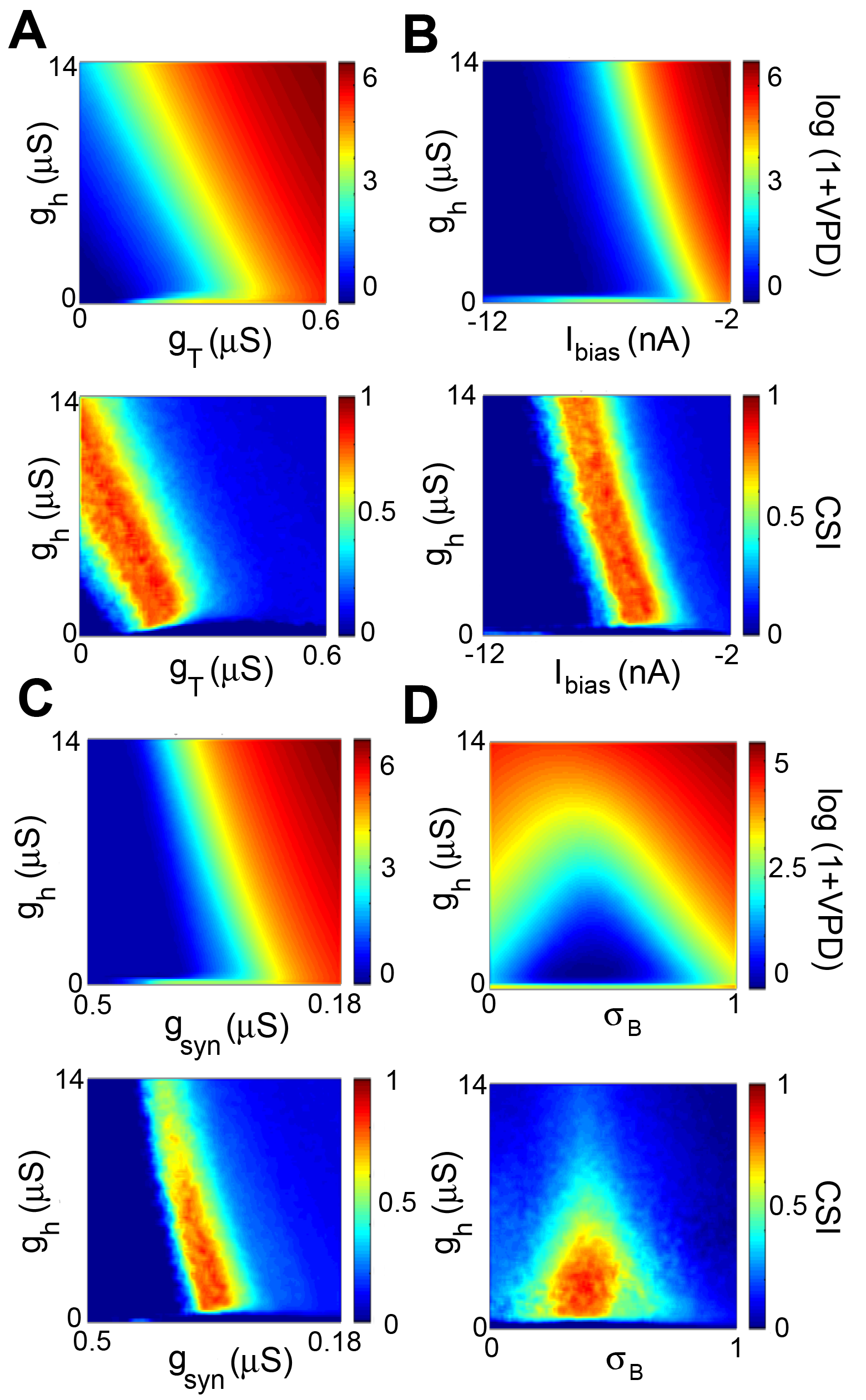

Supplement: S2 Fig — Parameter values are the same as in Fig 6. (TIF) [file pcbi.1004430.s002.tif]
